# Polyglutamine Expansion Accelerates the Dynamics of Ataxin-1 and Does Not Result in Aggregate Formation

**DOI:** 10.1371/journal.pone.0001503

**Published:** 2008-01-30

**Authors:** Hilde A. Krol, Przemek M. Krawczyk, Klazien S. Bosch, Jacob A. Aten, Elly M. Hol, Eric A. Reits

**Affiliations:** 1 Department of Cell Biology and Histology, Academic Medical Centre, University of Amsterdam, Amsterdam, The Netherlands; 2 Netherlands Institute for Neuroscience, Royal Netherlands Academy of Arts and Sciences Amsterdam, Amsterdam, The Netherlands; National Institutes of Health, United States of America

## Abstract

**Background:**

Polyglutamine expansion disorders are caused by an expansion of the polyglutamine (polyQ) tract in the disease related protein, leading to severe neurodegeneration. All polyQ disorders are hallmarked by the presence of intracellular aggregates containing the expanded protein in affected neurons. The polyQ disorder SpinoCerebellar Ataxia 1 (SCA1) is caused by a polyQ-expansion in the ataxin-1 protein, which is thought to lead to nuclear aggregates.

**Methodology/Principal Findings:**

Using advanced live cell fluorescence microscopy and a filter retardation assay we show that nuclear accumulations formed by polyQ-expanded ataxin-1 do not resemble aggregates of other polyQ-expanded proteins. Instead of being static, insoluble aggregates, nuclear accumulations formed by the polyQ-expanded ataxin-1 showed enhanced intracellular kinetics as compared to wild-type ataxin-1. During mitosis, ataxin-1 accumulations redistributed equally among daughter cells, in contrast to polyQ aggregates. Interestingly, polyQ expansion did not affect the nuclear-cytoplasmic shuttling of ataxin-1 as proposed before.

**Conclusions/Significance:**

These results indicate that polyQ expansion does not necessarily lead to aggregate formation, and that the enhanced kinetics may affect the nuclear function of ataxin-1. The unexpected findings for a polyQ-expanded protein and their consequences for ongoing SCA1 research are discussed.

## Introduction

Polyglutamine (polyQ) expansion disorders include diseases such as Huntington's disease (HD), dentatorubropallidoluysian atrophy (DRPLA), spinobulbar muscle atrophy (SBMA) and the spinocerebellar ataxias (SCA) type 1, 2, 3, 6, 7 and 17. These polyQ disorders are caused by an expansion of the CAG-trinucleotide repeat region in the respective disease-related genes. Although the different polyQ proteins are widely expressed in cells throughout the brain, there is a high variability in the cell type loss in different brain areas. In most polyQ disorders the disease becomes manifested when the polyQ expansion exceeds 36–40 glutamines. The length of the polyQ expansion is inversely correlated with the age of onset of the disease. All polyQ disorders are dominantly inherited and the general concept is that the disease is caused by a toxic gain of function of the polyQ-expanded protein. Furthermore, commonly for all polyQ disorders, the affected cells show intracellular aggregates containing the polyQ-expanded protein. This aggregation is a result of the extended polyQ stretch in the proteins. It is still unclear whether the aggregates are toxic for cells, as a protective role has also been suggested [1]–[4].

SCA1 is a polyQ disorder caused by a glutamine expansion in the protein ataxin-1, which results in selective loss of Purkinje cells in the cerebellum, atrophy of specific brain stem neurons and extensive loss of motor neurons in the spinal cord [5]. Patients suffer from progressive loss of motor coordination, speech impairment and problems with swallowing. In healthy individuals the levels of ataxin-1 expression in the central nervous system is two to four-fold of that in peripheral tissues [6]. The function of ataxin-1 is still elusive. Wild-type ataxin-1 is a nuclear protein that can shuttle between the nucleus and the cytoplasm [7]. In the Purkinje cells and brain stem neurons, ataxin-1 is mainly present in the nucleus and only to some extent in the cytoplasm. This is in contrast with the localization of the protein in non-neuronal tissues, where ataxin-1 is mainly in the cytoplasm [6], [8]–[10]. This suggests that the nuclear localization of ataxin-1 in Purkinje cells may contribute to the selectivity of the disorder. Indeed, transgenic mice expressing polyQ-expanded ataxin-1 with a mutated nuclear localization signal did not develop the disease, demonstrating that nuclear localization is critical for the pathogenesis [3].

While the function of ataxin-1 is still elusive, it has been suggested that ataxin-1 is involved in gene expression regulation, as it can bind to RNA and interact with various transcription factors [11]–[16]. Ataxin-1 contains an AXH (Ataxin-1 and HMG-box protein 1) domain that has been shown to interact with other proteins and RNA and that has been implicated to play a role in transcriptional repression [12], [16]–[20]. In addition, ataxin-1 has a self associating region spanning the amino acids 570 to 605 of the wild-type protein. This region overlaps partly with the AXH domain [19]. The presence of nuclear aggregates in the cerebellum of SCA1 patients has led to the assumption that the polyQ-expansion causes ataxin-1 to misfold and form intranuclear aggregates. Not only may these aggregates lead to neuronal toxicity, polyQ-expansion may also alter the normal function of ataxin-1, or lead to the loss of nucleocytoplasmic shuttling ability. While aggregates composed of polyQ-expanded proteins are generally static structures comprised of tightly aggregated proteins, we state that this assumption needs to be reevaluated in the case of SCA1.

Here we show that the kinetics of nuclear polyQ-expanded ataxin-1 accumulations are very different from other polyQ proteins. Both wildtype (Atx1[Q2]GFP) and polyQ-expanded (Atx1[85Q]GFP) accumulations turned out to be soluble, whereas other polyQ-GFP aggregates form insoluble structures. In addition, mitotic cells redistributed the nuclear accumulations of polyQ-expanded ataxin-1 proteins to both daughter cells, whereas ‘true’ polyQ aggregates were all trans-located to one daughter cell. In contrast to an earlier report [7], the polyQ-expansion did not affect shuttling of ataxin-1 between the nucleus and cytoplasm. Surprisingly, a longer polyQ-expansion led to an increase in speed of exchange of ataxin-1 between the nuclear accumulations and the free nuclear pool. In addition, we observed that the ataxin-1 accumulations were mobile and frequently fused with each other, and polyQ-expansion led to an increase in both mobility and fusion of the nuclear accumulations.

## Results

### PolyQ-expanded ataxin-1 does not form insoluble aggregates

**Figure 1:**
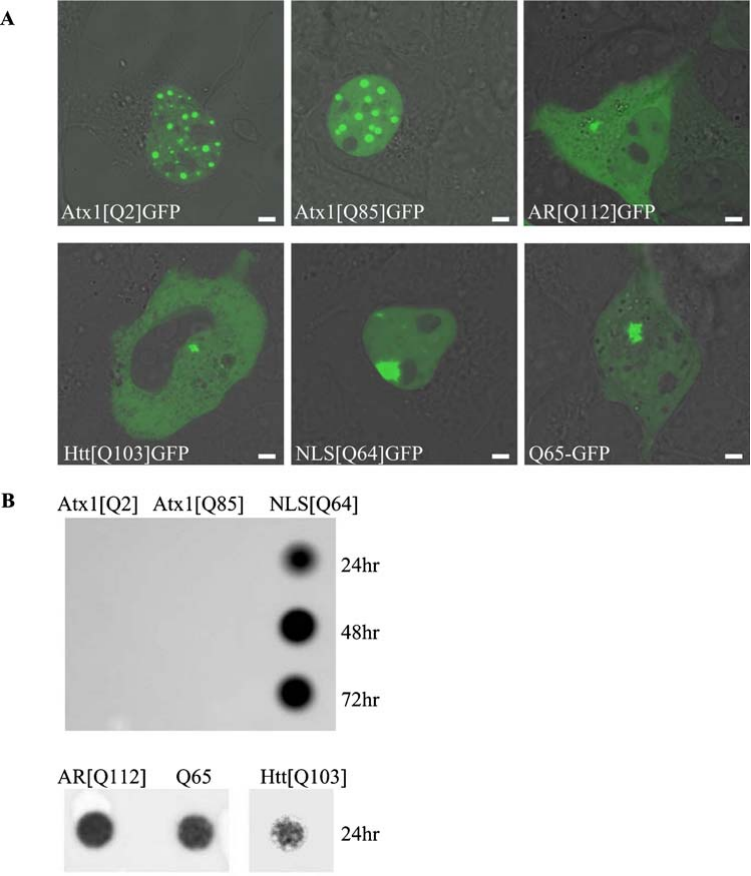
Nuclear ataxin-1 accumulations do not resemble aggregates formed by other disease-related polyglutamine-expanded proteins. (A). Live cell images of wild-type and polyQ-expanded ataxin-1 (Atx1[Q2]GFP and Atx1[Q85]GFP) and polyglutamine-expanded proteins androgen receptor (AR[Q112]GFP), huntingtin exon-1 (Htt[Q103]GFP), NLS[Q64]GFP and Q65-GPF in Cos7 cells. (B). Filtertrap assay of celllysates of Cos7 cells expressing Atx1[Q2]GFP, Atx1[Q85]GFP and NLS[Q64]GFP, 24, 48 and 72 hr after transfection (upper panel) and of Cos7 cells expressing AR[Q112]GFP, Q65-GFP and Htt[Q103]GFP 24 hours after transfection (lower panel). Sizebar = 1 µm.

PolyQ disorders show accumulation of polyQ-expanded proteins into a single cytoplasmic or nuclear aggregate. In agreement with data published previously [13], [21] our experiments demonstrated that ataxin-1 is mostly accumulating into multiple nuclear accumulations and this process is independent of the length of the polyQ expansion (Fig 1A). To compare the distribution and aggregate formation of ataxin-1 to a variety of different polyQ-expanded proteins we transfected Cos-7 cells with different polyQ proteins tagged with green fluorescent protein (GFP), to enable visualization in living cells. Cos-7 cells were chosen since they have a low expression level of endogenous ataxin-1 [8]. This minimizes interactions between the transfected ataxin-1 fusion proteins and the endogenous wild-type ataxin-1, thereby preventing any additional effect on the attaxin-1 aggregate formation. Next to the wildtype ataxin-1 (Atx1[2Q]GFP) and the polyQ-expanded ataxin-1 (Atx1[85Q]GFP), two disease-related polyQ-expanded fusion proteins were used, i.e. the truncated androgen receptor (AR[Q112]GFP) and huntingtin exon1 (Htt[Q103]GFP) which are both aggregation-prone (Fig 1A). We also expressed a pure polyQ-tract fused to GFP (Q65-GFP) and a polyQ-GFP fused to a nuclear localization signal (NLS[Q64]GFP). These fusion proteins are also aggregation-prone due to a similar polyQ-expansion. The NLS signal targets the protein to the nucleus, which mimics ataxin-1 polyQ localization. When cells were analyzed by Confocal Laser Scanning Microscopy, all polyQ-expanded proteins formed irregularly shaped intracellular aggregates within 24 hours after transfection, with exception of Atx1[85Q]GFP and Atx1[2Q]GFP which formed multiple nuclear accumulations (Fig 1A). The formation of multiple nuclear accumulations of Atx1[2Q]GFP has been described before [13], [21]. The distinct pattern of the formation of multiple nuclear accumulations is probably due to features of the ataxin-1 protein, since nuclear polyQ-expanded GFP (NLS[Q64]GFP) resulted in a single nuclear aggregate which increased in size in time due to sequestering of newly synthesized polyQ-GFP proteins, similar as observed with other polyQ-expanded proteins. This main difference between Atx1[85Q]GFP and NLS[Q64]GFP was maintained up to 72 hours after transfection.

As polyQ-protein aggregates form insoluble inclusions we examined whether the distinct difference in distribution between ataxin-1 and other polyQ proteins is also reflected by a difference in the solubility of the aggregates. By using a filter retardation assay, SDS-insoluble aggregates can be detected and quantified on a membrane while the remaining cell lysate passes through [22]. The solubility of both the Atx1[Q2]GFP and Atx1[85Q]GFP ataxin-1 was compared to NLS[Q64]GFP at three different time points after transfection (24, 48 and 72 hours, Fig 1B). At these timepoints most cells contained both small and large accumulations of Atx1[2Q]GFP, Atx1[85Q]GFP and NLS[Q64]GFP. Aggregates formed by the truncated androgen receptor (AR[Q112]GFP), Q65-GFP and huntingtin exon-1 (Htt[Q103]GFP) were already present after 24 hours and also varied in size from small to large. Figure 1B clearly shows that the ataxin-1 accumulations are soluble, whereas all other polyQ aggregates were trapped on the filter. This suggests that nuclear accumulations consisting of polyQ-expanded ataxin-1 cannot be defined as aggregates, as ataxin-1 accumulations are soluble and not sequestered into a single aggregate as generally observed in polyQ disorders. Instead of calling them aggregates, the term nuclear accumulations will now be used for these soluble structures.

### PolyQ expansion increases nuclear dynamics of ataxin-1

**Figure 2:**
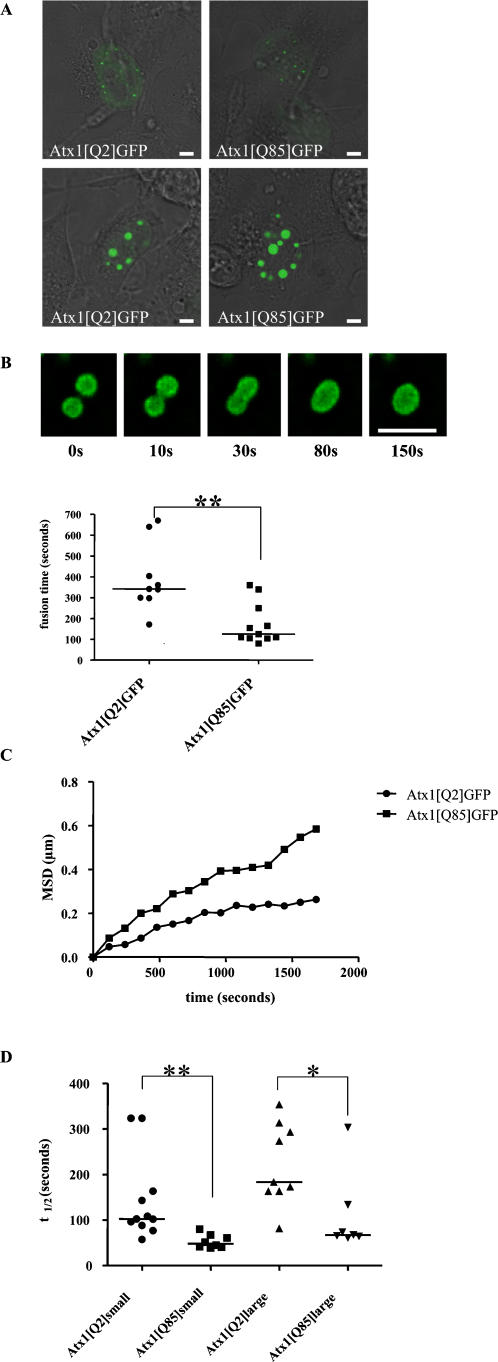
Polyglutamine expansion accelerates ataxin-1 dynamics. (A). Atx1[Q2]GFP and Atx1[Q85]GFP form similarly sized nuclear accumulations including small nuclear accumulations (upper panel) and large nuclear accumulations (lower panel). (B). PolyQ expansion enhances ataxin-1 nuclear accumulation fusion speed. Pictures 1–5 show fusion of two Atx1[Q85]GFP nuclear accumulations within 150 seconds. The fusion speed of Atx1[85Q]GFP nuclear accumulations is faster than of similarly sized Atx1[Q2]GFP nuclear accumulations (lower panel, n = 9 resp. 11). Data have been tested non-parametrically (**P<0.005). (C). Increased mobility of Atx1[Q85]GFP over Atx1[Q2]GFP nuclear accumulations 24 hours after transfection. The Y-axis depicts the average mean square displacement (MSD) of 93 Atx1[Q2]GFP nuclear accumulations and 128 similarly sized Atx1[Q85]GFP nuclear accumulations. (D). Atx1[Q85]GFP nuclear accumulations have a higher on/off rate than Atx1[Q2]GFP nuclear accumulations. FRAP live cell experiments were performed 24 hr after transfection. Small and large nuclear accumulations have been analyzed separately. Half-time recovery data have been tested non-parametrically (**P<0.005, *P<0.05). Sizebar = 1 µm.

Since polyQ aggregates are generally static structures characterized by a low on/off rate of the aggregated proteins, we next examined whether the length of the polyQ-expansion would affect the kinetics of the nuclear ataxin-1 accumulations. To compare the kinetics in a background of low endogenous ataxin-1 levels we transfected both Atx1[Q2]GFP and Atx1[Q85]GFP into Cos-7 cells and analyzed formed nuclear accumulations by confocal microscopy. Both Atx1[Q2]GFP and Atx1[Q85]GFP formed nuclear accumulations of various sizes, and we observed movement and fusion of all types of nuclear accumulations in time (Fig 2A, supplementary Movie S1). We next investigated whether the polyQ length might affect the duration of the fusion in the cell nucleus by determining the time needed for a complete fusion event. Time-lapse imaging monitored the fusion process of similarly sized nuclear ataxin-1 accumulations. As shown in Figure 2B and Movie S1 in the supplementary material, the fusion of two nuclear Atx1[Q85]GFP accumulations is faster than of two similarly sized Atx1[Q2]GFP accumulations (P = 0.0019). We analyzed respectively 9 and 11 fusion events for Atx1[Q2]GFP and Atx1[Q85]GFP in individual cells and we observed that the fusion of the nuclear Atx1[Q2]GFP accumulations was finished in a time between 172 and 670 seconds (median = 342 seconds) and of the Atx1[Q85]GFP accumulations between 80 and 340 seconds (median = 125 seconds). This suggests that the nuclear accumulations of Atx1[Q85]GFP are more dynamic then Atx1[Q2]GFP, and that polyQ expansion accelerates the fusion between nuclear ataxin-1 accumulations. In addition we studied whether the Atx1[Q2]GFP and Atx1[Q85]GFP accumulations have different mobilities in the nucleus. We compared the mobility of equally sized accumulations of Atx1[Q2]GFP or Atx1[Q85]GFP in the nucleus 24 hours after transfection. The mobility of 93 nuclear Atx1[Q2]GFP accumulations, measured in 12 individual cells, was compared to 128 Atx1[Q85]GFP accumulations in 16 individual cells. The mean square displacement of these accumulations has been calculated after each time point. The presence of a polyQ expansion resulted in an increased mobility of the nuclear accumulations in the nucleus (Fig 2C). The accelerated fusion speed and mobility of Atx1[Q85]GFP contrasts with the general view that polyQ aggregates are static, immobile structures composed of irreversibly trapped polyQ proteins.

To examine the on/off rate of nuclear ataxin-1 accumulations we performed Fluorescence Recovery after Photobleaching (FRAP) experiments to determine the exchange of ataxin-1 present between accumulations and the nuclear environment. Using FRAP, irreversible photobleaching leads to permanent loss of the fluorescence of the selected nuclear ataxin-1 accumulation, and fluorescence recovery can only occur when bleached ataxin-1 is replaced by fluorescent ataxin-1 from other nuclear accumulations or the free pool. The time needed for the recovery of fluorescence is therefore representative for the rate of exchange between the bleached nuclear accumulation, the surrounding Atx1-GFP fluorescent accumulations and the nuclear pool of Atx1-GFP. Surprisingly, when measuring the t_1/2 _(which is the time point where the fluorescence has recovered to 50% of its original fluorescence intensity level), we found that fluorescence of the Atx1[Q85]GFP accumulations recovered much faster than Atx1[Q2]GFP (Fig 2D). In addition, we measured differences in the recovery of the small and large nuclear accumulations, since we anticipated that larger accumulations would show a lower exchange due to the reduction in surface/volume ratio. The t_1/2 _of small Atx1[Q85]GFP accumulations (median = 48 seconds) was significantly lower than the t_1/2 _of small Atx1[Q2]GFP accumulations (median = 102.4 seconds) (P = 0.001), measured in respectively 8 and 11 bleaching experiments in individual cells. In addition, the t_1/2 _of large Atx1[Q85]GFP accumulations (median = 67.2 seconds) was also significantly lower than Atx1[Q2]GFP accumulations (median = 183.6 seconds) (P = 0.013), measured in respectively 7 and 9 experiments. We observed no difference in size between the immobile fractions of the nuclear accumulations formed by either Atx1[Q2]GFP or Atx1[Q85]GFP (data not shown). These data indicate that polyQ-expansion enhances the kinetics of ataxin-1, leading to less stable structures, which is also suggested by the accelerated fusion speed.

### Nuclear ataxin-1 accumulations separate symmetrically during cell division

**Figure 3:**
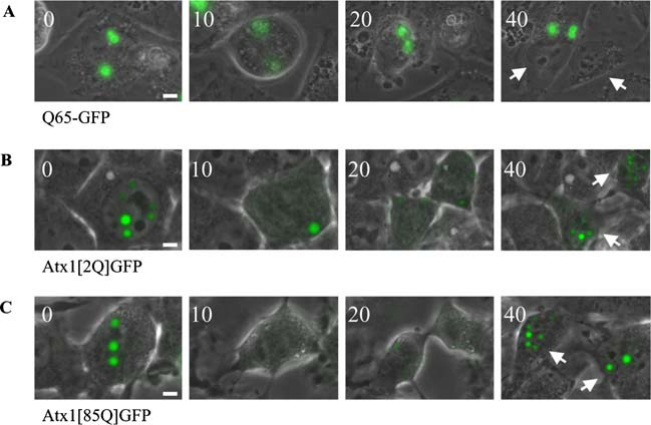
Nuclear ataxin-1 accumulations distribute symmetrically to daughter cells during cell division. Representative series of images showing cell division of a cell containing fluorescent aggregates or nuclear accumulations. (A). Asymmetric distribution of Q65-GFP aggregates in time. Arrows indicate the two daughter cells. (B). Symmetric distribution of Atx1[Q2]GFP nuclear accumulations during cell division. Nuclear accumulations fuse prior to division. After division both daughter cells contain cytoplasmic accumulations and later nuclear accumulations. (C). Symmetric distribution of Atx1[Q85]GFP nuclear accumulations during cell division. Time is indicated in minutes. Sizebar = 1 µm.

Cells containing polyglutamine aggregates are still able to enter mitosis [23] (and unpublished observation). Interestingly, when cells contained multiple aggregates, these aggregates were always distributed to only one of the daughter cells, whereas the other daughter cell was free of any aggregate. We therefore examined whether polyQ-expanded ataxin-1 accumulations are also asymmetrically divided during mitosis, by following the nuclear accumulations in mitotic cells that expressed either Q65-GFP, Atx1[Q2]GFP or Atx1[Q85]GFP, using automated time-lapse fluorescence imaging. Indeed, mitotic cells containing multiple polyQ nuclear aggregates formed by Q65-GFP showed asymmetrical separation of aggregates to only one daughter cell (Fig 3A). In contrast, cells containing multiple nuclear accumulations formed by either Atx1[Q2]GFP or Atx1[Q85]GFP distributed ataxin-1 to both daughter cells, regardless of polyQ expansion (Fig 3B,C). Note that most nuclear accumulations fuse into large accumulations shortly before entering mitosis, while right after cell division, both daughter cells again contain multiple small nuclear accumulations.

### PolyQ expansion does not affect ataxin-1 nuclear shuttling

**Figure 4:**
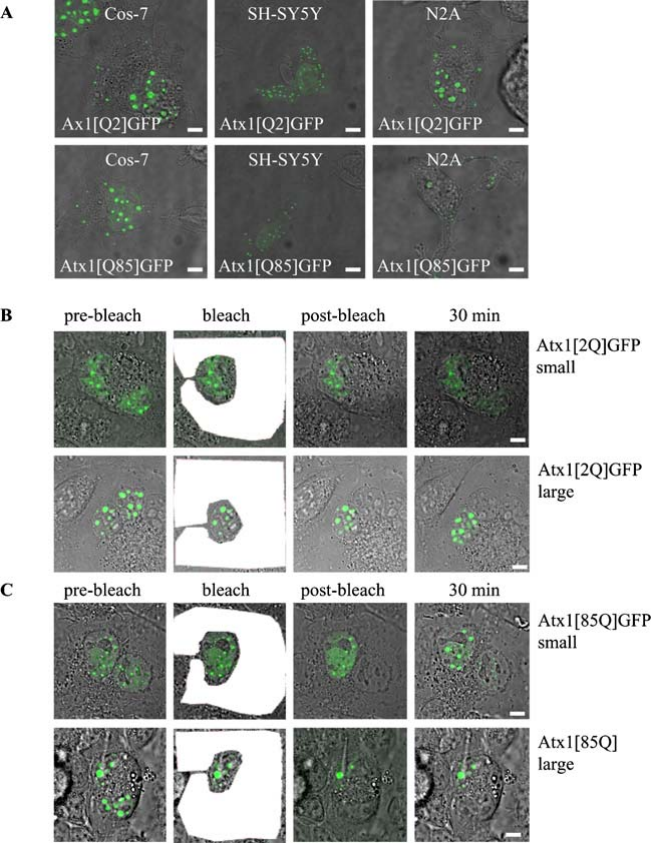
Polyglutamine expansion does not affect ataxin-1 shuttling between the nucleus and the cytoplasm. (A). Cos-7 cells and N2A and differentiated SH-SY5Y neuronal cells contain cytoplasmic accumulations of both Atx1[Q2]GFP and Atx1[Q85]GFP. (B). Representative nucleocytoplasmic shuttling assay in a Cos-7 bikaryon with small and large Atx1[Q2]GFP nuclear accumulations. Shuttling to the bleached nucleus is only observed when small nuclear accumulations are present. (C). Atx1[Q85]GFP can shuttle between nucleus and cytoplasm when bikaryons of Cos-7 cell contain small nuclear accumulations. When large nuclear accumulations are present there is no nucleocytoplasmic shuttling of either Atx1[Q2]GFP or Atx1[Q85]GFP. The bleached region is indicated in white. Sizebar = 1 µm.

As wild-type ataxin-1 can shuttle between the nucleus and the cytoplasm of a cell, it has been suggested that ataxin-1 is involved in RNA binding and transport to the cellular periphery [7]. This transport may be abolished in SCA1, as polyQ expansion hampers the nuclear export of ataxin-1 [7]. It should however be noted that in this study small wild-type ataxin-1 nuclear accumulations were compared to large polyQ-expanded ataxin-1 nuclear accumulations. As we showed that large accumulations of both polyQ-expanded Atx1[Q85]GFP and wildtype Atx1[Q2]GFP have a lower recovery rate when compared to small accumulations this might have limited the exit of free ataxin-1 from the nucleus. We also observed that Cos-7 cells expressing either Atx1[Q2]GFP or Atx1[Q85]GFP showed cytoplasmic presence of the protein, either diffuse or in body-like structures, independent of the length of the polyQ expansion (Fig 4A). The presence of cytoplasmic polyQ-expanded ataxin-1 is not limited to Cos-7 cells, as cultured neuroblastoma cells such as SH-SY5Y and N2A neuroblastoma cells (Fig 4A) as well as U343MG astrocytoma cells (data not shown) showed cytoplasmic presence and movement of ataxin-1 accumulations. The cytoplasmic presence may reflect the suggested role of ataxin-1 in RNA binding and shuttling to cytoplasmic domains [7]. To test whether polyQ-expansion limits the nuclear-cytoplasmic shuttling of ataxin-1, we performed a nucleocytoplasmic shuttling assay with Atx1[Q2]GFP and Atx1[Q85]GFP transfected in Cos-7 cells. To measure the nuclear shuttling, we performed FRAP analysis on cells containing two nuclei (bikaryons) by photobleaching all fluorescence except for one ataxin-1 nucleus and visualized the exchange and shuttling of GFP-tagged ataxin-1 by imaging the increase of fluorescence in the bleached nucleus. These experiments were performed in the presence of the translation inhibitor cycloheximide to prevent fluorescence recovery due to de novo synthesis of ataxin-1. We distinguished between cells having either only small or large nuclear ataxin-1 accumulations. Cells having only free nuclear ataxin-1 distribution or ataxin-1 present in small nuclear accumulations showed recovery of fluorescence in the bleached nucleus within 30 minutes after bleaching, independent of the length of a polyQ expansion (Fig 4B,C, upper panel). However, the bikaryons with large nuclear ataxin-1 accumulations did not show any recovery within this time span. This observation was independent of the length of the polyQ tract (Fig 4B,C lower panel). Taken together, these data show that the shuttling of ataxin-1 only occurs when the protein is diffusely dispersed in the nucleoplasm or when the protein is present in small nuclear accumulations. More importantly, the ability of ataxin-1 to shuttle from the nucleus is not affected by the length of the polyQ expansion.

## Discussion

PolyQ disorders are characterized by the presence of insoluble, intracellular aggregates initiated by the respective polyQ-expanded proteins, and these aggregates are also present when the polyQ expansion is fused to non disease-related proteins (e.g. Q65-GFP). Nuclear aggregates are present in neurons of several brain areas in transgenic mice expressing an expansion of 146 glutamines fused to a non disease-related protein [24]. While processes like autophagy may clear the cytoplasm from polyQ aggregates, the nucleus seems particularly sensitive since in all polyQ diseases the aggregates have been primarily found in the nuclei of patient material tissue [25]. It is therefore not surprising that SCA1 is often categorized as one of the polyQ disorders due to the presence of nuclear aggregates. However, various reports have shown that polyQ expansion is not the only domain which can induce ataxin-1 accumulation as the self associating region and two other domains seem to be involved in ataxin-1 self-association [12], [19]. Accordingly, wild-type ataxin-1 can also form similar nuclear structures, and deletion of the self associating region prevents nuclear accumulation of polyQ-expanded ataxin-1 in COS cells and transgenic mice [3], [10]. This is in contradiction with findings showing that polyQ expansion leads to inclusion formation in SCA1.

We observed that nuclear polyQ-expanded ataxin-1 accumulations could not be detected by a filter retardation essay, whereas other polyglutamine aggregating proteins where clearly insoluble (including nuclear polyQ-GFP proteins). The non-static behavior of nuclear Atx1[Q85]GFP accumulations was also shown by their ability to move and fuse within the nucleus, with a surprising increase in the fusion speed and on/off rates as compared to wild-type nuclear ataxin-1 (Atx1[Q2]GFP) accumulations. The enhanced fusion could also result in a slightly higher amount of large nuclear accumulations in time in Atx1[Q85]GFP cells [21]. The enhanced on/off rate of polyQ-expanded ataxin-1 is different from earlier observations by Stenoien and colleagues, who showed that polyQ expansion of ataxin-1 leads to reduced exchange within nuclear accumulations [21]. Since ataxin-1 has been shown to associate with the nuclear matrix [8], structures incorporating polyQ-expanded ataxin-1 may be less stably associated with the matrix then the wild-type ataxin-1, which might in turn lead to an increase of their mobility. However the nuclear matrix is a structure that is not clearly defined and it might be more appropriate to suggest that ataxin-1 associates with the nuclear scaffold. It has been suggested that ataxin-1 is present in a transcription/RNA processing complex [7], [18] whose functionality is lost upon transcription inhibition. An example is the known complex of ataxin-1, RORα, a transcription factor critical for cerebellar development, and tip60, a co-activator of RORα [26], [27]. The polyglutamine expansion might not only affect the self-association of ataxin-1, but may also disturb the interaction with proteins such as RORα and tip60, resulting in alteration of transcriptional activity of several proteins and downregulation of important proteins [28]. The observed decrease in stable complex interactions of polyQ-expanded ataxin-1 containing nuclear accumulations might therefore affect its regulatory function.

Besides enhanced kinetics and solubility, a third major difference between polyQ-expanded ataxin-1 and other polyQ proteins was the separation of aggregates during cell division. Cells containing multiple polyQ aggregates are viable enough to enter mitosis, and by yet undefined mechanism all aggregates segregate into one daughter cell. While it is attractive to think that this might be a mechanism allowing separation of harmful proteins into one cell and leaving the other free of aggregates, it is of less importance for neurons, most of which are post-mitotic. In contrast to the asymmetrically dividing polyQ aggregates, nuclear polyQ ataxin-1 accumulations are redistributed equally among the daughter cells. They fuse into large accumulations prior to the actual M phase, and during the actual division the accumulations rapidly dissociate into a diffuse nuclear distribution. As the appearance of small accumulations can be observed soon after cell division, this mechanism may guarantee equal distribution of these proteins to both daughter cells. This phenomenon also occurs in case of specific cytoplasmic organelles but also the nucleoli (which also has a function in local transcription and RNA binding), resulting in equal redistribution to both daughter cells [29]. It also supports our model that nuclear accumulations formed by ataxin-1 resemble functional complexes and not aggregates. Therefore we suggest that ataxin-1 nuclear bodies should be a better definition than aggregates, inclusion bodies or accumulations

Shortly after mitosis we observed a rapid re-formation of multiple ataxin-1 nuclear bodies that subsequently localize to the newly formed nucleus. The rapid ataxin-1 dynamics and redistribution underscores our hypothesis that polyQ-expanded ataxin-1 is able to shuttle through the nuclear pore complex and is in contrast by findings from Irwin and collegues [7]. In this study the nucleocytoplasmic shuttling capability of wild type ataxin-1 in cells containing small nuclear bodies was compared with cells containing only large polyQ-expanded ataxin-1 nuclear bodies. Since the size of the nuclear bodies affects on/off ratios and therefore the amount of free ataxin-1, we compared similarly-sized nuclear bodies between Atx1[Q2]GFP and Atx1]Q85]GFP expressing cells. Interestingly, if only large nuclear bodies are present there is no nucleocytoplasmic shuttling of either ataxin-1 protein. In the case of free nucleoplasmic distribution or only small nuclear bodies, shuttling is not impaired by polyQ expansion. To unravel SCA1 disease it will be important to understand the nature of these nuclear bodies and to examine their composition and function in both the nucleus and cytoplasm But if the polyQ expansion does not induce aggregation or impair nuclear shuttling, what may be affected in SCA1? Ataxin-1 is involved in multiple pathways as suggested previously and each of these might contribute to SCA1 pathogenesis [26]. PolyQ-expanded ataxin-1 might play its role at the transcriptional level and alter the transcription of genes important for Purkinje cells [11], [26], [28]. In addition polyQ-expanded ataxin-1 could be involved in post-transcriptional processes such as mRNA splicing through its interaction with polyglutamine-tract-binding protein 1 (PQBP1) [14], a protein that has been shown to interact with the splicing factor SIPP1 [30]. PolyQ expansion of ataxin-1 also influences its binding to RNA [18] and in this manner could alter its suggested role in nucleocytoplasmic shuttling of mRNA [7], a process that is very important for local translation of proteins in neurons.

## Materials and Methods

### DNA constructs

Q65-GFP was generated using Ub-M-GFP-Q65 as a template (kindly provided by Nico Dantuma, Stockholm, Sweden) using primers flanking the polyQ stretch (forward: CCGGAATTCACCATGGAGTACACACCTCCCGGCGCCAGTTT with EcoRI site and reverse: GGATCCCGGGCCCCTCCTGGGGCTAGTCTCTTGCTG with ApaI site) and subsequent ligation into EGFP-N3 vector (Clontech, Palo Alto, CA). Htt[Q103]GFP was kindly provided by Ron Kopito, Stanford, California. AR[Q112]GFP contains a truncated version of the androgen receptor tagged with GFP (kindly provided by Paul Taylor, Philadelphia, Pennsylvania). The NLS[Q64]GFP contains a nuclear localization signal (NLS) to target the protein to the nucleus (kindly provided by Itaru Toyoshima, Akita, Japan). Atx1Q2]GFP and Atx1[Q85]GFP contain full length ataxin-1 cloned into a pEGFP-C2 vector (kindly provided by Huda Zoghbi, Houston, Texas).

### Cell culture and transfection

Cos7, SH-SY5Y and N2A cells were cultured in Dulbecco's Modified Eagle Medium (DMEM) supplemented with 10% fetal bovine serum and 5% penicillin (100 U/ml), streptomycin (100 mg/ml) and glutamine (100 mg/ml). U343MG astrocytomas cells were cultured in DMEM (high glucose) and HAM F10 (1∶1) supplemented with 10% fetal bovine serum, 5% penicillin and 100 U/ml streptomycin. Cells were maintained at 37°C in an atmosphere of 5% CO_2_. For live cell microscopy 0.2×10^−6^ cells were plated on glass coverslips (24 mm; Fisher scientific, Braunsweg, Germany) in a 6 well plate and transfected with DNA plasmids after 24 hours using Lipofectamine 2000 transfection reagent according to manufacturer's instructions (Invitrogen) for Cos-7, N2A and U343MG astrocytoma cells. SH-SY5Y cells were transfected using dreamfect gold (OZ Biosciences). Retinoic acid (Sigma R 2625, final concentration 10 µM) was added to the SH-SY5Y culture medium for 3 days to differentiate them. For fixation 4% paraformaldehyde in PBS was used.

### Filter retardation assay

Filter retardation assay was performed as described before [22]. Briefly, cell lysates were incubated for 30 minutes on ice and centrifuged at 14,000 rpm for 15 minutes at 4°C. Pellets containing the insoluble material and aggregates were resuspended in benzonase buffer (1 mM MgCl_2_, 50 mM Tris-HCl; pH 8.0) and incubated for 1 hour at 37°C with 1 µl benzonase (100,000 units/vial (Merck, Darmstadt, Germany). Incubations were terminated by adding 100 µl 2x termination buffer (40 mM EDTA, 4% SDS, 100 mM DTT). Equal amounts of protein extracts were diluted in 200 µl 2% SDS and filtered on a dot-blot filtration unit through a cellulose acetate membrane (Schleicher and Schuell, 0.2-µm pore size) that has been pre-equilibrated with 2% SDS. Filters were washed twice with 200 µl 0.1%SDS and GFP fluorescence was measured by using the LAS-3000 (Fujifilm).

### Confocal Laser Scanning Microscopy (CLSM) and Fluorescence Recovery After Photo-bleaching (FRAP) analysis

Transfected cells were categorized by the size of the nuclear accumulations: small (≤1 µm diameter) and large (≥2.5 µmsize). FRAP analysis was performed using a Leica Sp2 CLSM adapted for living cell analysis using a 63x oil immersion objective. A selected ataxin-1 accumulation was repeatedly bleached in 10 frames at maximum laser power, resulting in a reduction of fluorescence to less than 10% of the initial value. Fluorescence recovery was measured by time-lapse imaging for 10 minutes post bleaching. Intranuclear mobility of the nuclear accumulations in time was corrected for overall movement of the nucleus by a custom-written software based on Matlab (Mathworks, Inc., USA) DipImage and IterativeClosest Point algorithm (Krawczyk, P in press).

### Nucleocytoplasmic transport assay

Cells were incubated with cycloheximide (final concentration 200 mM) for 15 minutes prior to cell fusion, which was performed by washing the cells with PBS, incubating them in 50% (w/v) polyethylene glycol 1500 (Roche Molecular Biochemicals) for 2 min, and rinsing with PBS. Cells were then incubated for 20 minutes in the cell culture medium containing 100 µg/ml cycloheximide before analysis by CLSM. During the experiment the cells were incubated in the same cell culture medium including cycloheximide. FRAP was performed on a laser scanning confocal microscope (Leica Sp2 CLSM) by photobleaching a region surrounding the bikaryon but one nucleus. Recovery of fluorescence in the bleached nucleus was monitored in time and compared to the fluorescence intensity of the second nucleus in the bikaryon.

### Life cell imaging

Time lapse movies were performed using a Leica (DM-IRBE) inverted microscope and a 63x oil immersion objective enclosed in a 37°C incubator with atmosphere of 5% CO_2_. Images were created using a GFP filter set (Leica c1) for GFP excitation. For the analysis of accumulation behavior during cell division fluorescent and phase contrast images have been taken every 10 minutes. For the analysis of accumulation mobility, 3-D, time-lapse movies of nuclear ataxin-1 accumulations, acquired every 2 min have been deconvolved and corrected for cell mobility during the experiment. Next, mean squared displacement (MSD) of the nuclear accumulations during 30 minutes was calculated. For the analysis of accumulation fusion, 2-D images were acquired every 2 seconds. Fusion was calculated as a time between touching of two fusing nuclear accumulations and rounding of the resulting accumulation.

## Supporting Information

Movie S1 Fusion of nuclear ataxin-1 accumulations. Time lapse movie of nuclear Atx1[Q85]GFP accumulations fusion. Time between two frames is 20 seconds.
(1.55 MB AVI)
